# Development of a digital GARD visual memory task to detect an early cognitive change in Alzheimer's disease

**DOI:** 10.1002/alz70856_102275

**Published:** 2025-12-25

**Authors:** Eun Hyun Seo, Hyung‐Jun Yoon, Seung Gon Kim, Kyu Yeong Choi, Sangsun Yi, Jahae KIM, Ho‐Chun Song, Ari Chong, Jung‐Min Ha, Sungho Won, Kun Ho Lee

**Affiliations:** ^1^ Premedical Science, College of Medicine, Chosun University, Gwangju, Korea, Republic of (South); ^2^ Gwangju Alzheimer's & Related Dementias (GARD) Cohort Research Center, Gwangju, Korea, Republic of (South); ^3^ College of Medicine, Chosun University, Gwangju, Korea, Republic of (South); ^4^ Department of Neuropsychiatry, Chosun University Hospital, Gwangju, Korea, Republic of (South); ^5^ Gwangju Alzheimer's and Related Dementia (GARD) Cohort Research Center, Chosun University, Gwangju, Korea, Republic of (South); ^6^ Hankyong National University, Anseong, Gyeonggi, Korea, Republic of (South); ^7^ Chonnam National University Medical School and Hospital, Gwangju, Korea, Republic of (South); ^8^ Department of Nuclear Medicine, School of Medicine, Chosun University/Chosun University Hospital, Gwangju, Korea, Republic of (South); ^9^ RexSoft Corps, Seoul, Korea, Republic of (South); ^10^ Gwangju Alzheimer's Disease and Related Dementia Cohort Research Center, Chosun University, Gwangju 61452, Korea, Republic of (South)

## Abstract

**Background:**

Detecting subtle cognitive changes in the very earliest stages of Alzheimer's disease (AD) is a crucial issue. Advancements in computer technology have increasingly integrated into neuropsychological assessments. This study aims to develop a visual memory paradigm on a tablet platform, designed to facilitate the early cognitive detection of AD.

**Method:**

The GARD visual memory test is based on the delayed‐matching‐to‐sample paradigm. The test includes three conditions: Color, Shape, and Color‐Shape. The development of test followed approach: (1) A cognitive neuroscience paradigm guided the design of an initial item pool. (2) Parallel test sets were developed to minimize learning effects. (3) Extensive user testing and collaboration with experts in elderly user experience. We included 131 cognitively normal (CN) and 71 cognitively impaired (CI) participants from the Gwangju Alzheimer's Disease and Related Dementia (GARD) Cohort in Korea. A battery of neuropsychological test scores and standardized uptake value ratios (SUVR) from amyloid PET scans were collected. We performed ANOVAs, regression, Intraclass correlation (ICC), and Pearson correlation analyses to examine the reliability and validity of the test.

**Result:**

All participants completed the visual memory test. All GARD visual memory test scores were significantly influenced by age and education, with no effects of gender observed. For reliability, ICC coefficients were significant across all test scores, ranging from 0.4 to 0.8. Regarding validity, visual memory scores were significantly correlated with paper‐and‐pencil measures of visual memory and global cognition. Time measurements showed significant correlations with paper‐and‐pencil psychomotor speed scores (all *p* < 0.001). All test scores were significantly different between CN and CI (all *p* < 0.001). Total and color score were negatively correlated, while Time measurements were positively correlated with SUVR.

**Conclusion:**

Our preliminary results suggest that the GARD visual memory test is a reliable and valid tool for assessing visuospatial memory and detecting early cognitive changes in AD. The GARD visual memory test shows promise as a sensitive tool for detecting cognitive changes in preclinical AD. Continued development and validation, including pathological biomarker studies and longitudinal research, will further establish its utility in clinical and research settings for early AD detection.

